# A Use of 56-kDa Recombinant Protein of *Orientia tsutsugamushi* Karp Serotype in Serodiagnosis of Scrub Typhus by Enzyme-Linked Immunosorbent Assay in Thais

**DOI:** 10.3390/tropicalmed8010010

**Published:** 2022-12-23

**Authors:** Phanita Chankate, Thareerat Kalambaheti, Nathamon Kosoltanapiwat, Ampai Tanganuchitcharnchai, Stuart D. Blacksell, Narisara Chantratita, Pornsawan Leaungwutiwong

**Affiliations:** 1 Department of Microbiology and Immunology, Faculty of Tropical Medicine, Mahidol University, Bangkok 10400, Thailand; 2 Mahidol-Oxford Tropical Medicine Research Unit (MORU), Faculty of Tropical Medicine, Mahidol University, Bangkok 10400, Thailand; 3 Centre for Tropical Medicine & Global Health, Nuffield Department of Medicine, University of Oxford, Oxford OX3 7LG, UK

**Keywords:** scrub typhus, *Orientia tsutsugamushi*, 56-kDa type-specific antigen, ELISA, recombinant protein, diagnostics

## Abstract

Scrub typhus is a mite-borne disease caused by a Gram-negative obligately intracellular bacillus, *Orientia tsutsugamushi*. The disease is endemic in the Asia–Australia–Pacific region, including Thailand. Scrub typhus generally manifests as acute undifferentiated febrile fever along with myalgia, rash, and lymphadenopathy. An eschar can be a valuable diagnostic clue, but this skin lesion may be missed in some patients. The disease symptoms resemble those of other febrile illnesses such as leptospirosis, typhoid, murine typhus, malaria, and dengue fever, making a laboratory diagnosis necessary for the definitive diagnosis. In this study, we expressed a recombinant protein derived from 56-kDa type-specific antigen of *O. tsutsugamushi* Karp serotype and tested its ability to detect and differentiate scrub typhus infection. IgM and IgG antibodies were determined in sera from scrub typhus (n = 92) and other febrile illness patients (murine typhus (n = 25), melioidosis (n = 36), leptospirosis (n = 42), and dengue (n = 35)) from Thailand. Sensitivities of 87.0% and 59.8% with a specified assay cut-off were obtained for IgM and IgG indirect ELISAs, respectively, with a specificity of 100% in both tests. The sensitivity was increased to 95.7% when a combination of IgM and IgG ELISAs results was considered. Our study suggested a potential of the 56-kDa recombinant protein for further development and evaluation for use in scrub typhus serodiagnosis.

## 1. Introduction

Scrub typhus, also known as tsutsugamushi disease, is caused by the arthropod-borne Gram-negative obligately intracellular bacillus, *Orientia tsutsugamushi* [1]. The disease has been reported worldwide, with the Asia–Australia–Pacific region (also known as the tsutsugamushi triangle), including Thailand, as an endemic area [2,3,4,5]. A rising number of reports among travelers returning to nonendemic countries makes this neglected disease a more interesting subject [4]. *Orientia tsutsugamushi* is transmitted to mammalian hosts, including humans, by the larval stage of trombiculid mites, i.e., *Leptotrombidium* sp. [6]. Larvae of the mites, also called chiggers, exhibit low host specificity and only feed once on a mammal host. The feeding lasts 2 to 4 days [7,8]. Chiggers enter the host skin via hair follicles or pores without piercing the host skin [8,9]. The mite saliva can dissolve host tissue around the feeding site, and the mites feed on the liquefied tissue. *Orientia tsutsugamushi* has been found in the salivary glands of mites [8,9]. A chigger acquires *Orientia* from an infected host during horizontal transmission, and its offspring infect a new host [8].

The first report of human scrub typhus in Thailand can be dated back more than five decades [10]. The national surveillance data collected during 2003–2018 revealed that the scrub typhus incidence in Thailand had risen significantly over the last two decades, with the majority of cases reported from provinces in northern and northeastern regions [11]. A high disease prevalence is associated with agricultural workers and people living in rural and mountainous areas [11]. Clinical presentations of scrub typhus in humans include fever, myalgia, rash, lymphadenopathy, and conjunctival injection [12]. In approximately 50% of patients, an eschar, which represents a site of a chigger bite, can be seen. Generally, diagnosis of scrub typhus is based on the clinical presentation and a patient’s history, with the presence of an eschar as a useful diagnostic clue. In the absence of an eschar, delayed diagnosis, complications, and high mortality may occur since the general manifestations of scrub typhus can resemble those in other febrile diseases such as leptospirosis, typhoid, murine typhus, malaria, dengue, and viral hemorrhagic fevers [4,12,13]. In cases with complications, the condition can involve multiple organ systems, resulting in pneumonitis, acute respiratory distress syndrome, acute kidney injury, myocarditis, meningoencephalitis, and hepatitis [4]. No reports of person-to-person transmission of scrub typhus exist [14]. Scrub typhus can be treated by antibiotics, generally doxycycline or chloramphenicol, although issues of antibiotic resistance are rising [2]. Median mortality of 6.0% and 1.4% were reported for untreated and treated scrub typhus, respectively [15]. No effective vaccine is available for scrub typhus; therefore, optimized treatments and diagnostic tests are still required.

Molecular and serological approaches can make a diagnosis of scrub typhus. The molecular techniques, i.e., PCR, real-time PCR, and LAMP for detection of *O. tsutsugamushi* genes, such as *56-kDa*, *47-kDa*, *groEL*, and *16S rRNA*, have been reported [16,17,18]. While those techniques generally show exceptionally high sensitivity and specificity, the issues of cost, technical expertise, and instrument requirement make the tests, i.e., PCR and real-time PCR, unsuitable in some endemic areas. LAMP or loop-mediated isothermal amplification has been developed to overcome those limitations. It is inexpensive, simple to perform, and requires only a common laboratory equipment such as a water bath or heating block [18]. Nevertheless, the sensitivity and specificity of molecular assays are found to vary depending on specimen type, period of specimen collection, and target genes [16]. Thus, serological detection is still widely used, and the combination of serological and molecular tests improve the diagnostic sensitivity [17]. IgM antibody detection can indicate recent infection using serological assays, while IgG detection is helpful in seroprevalence studies. Antibody detection for scrub typhus is generally done by indirect immunofluorescence (IFA), indirect immunoperoxidase (IIP), and enzyme-linked immunosorbent assay (ELISA). A rapid immunochromatography test was also developed for point-of-care diagnosis [2,19].

Serology for diagnosis of scrub typhus is limited by low sensitivity in the early disease course and by the requirement of paired samples. Still, it remains the mainstay for diagnosis because of its low cost and relative simplicity [20]. IFA and IIP tests provide satisfactory results in the hands of experienced persons but can be troublesome for inexperienced technicians because of microscopic evaluation [21]. The long-standing suboptimal gold standard IFA that requires a dynamic ≥four-fold rise in paired serum collections is notoriously difficult to standardize due to operator subjectivity and various local diagnostic cut-offs and requires improvement in terms of standardization and ease of use/throughput [22,23,24]. The antigens for assay, which are generally a whole cell preparation of the *O. tsutsugamushi* strains, Karp, Kato, and Gilliam, require propagation and purification in a biosafety level 3 (BSL3) facility, thus making more effort for a normal laboratory [19]. The use of a recombinant protein as an antigen in immunoassays can improve the issue of the BSL3 facility requirement. The recombinant 56-kDa major outer membrane protein of *O. tsutsugamushi* was reported to exhibit a sensitivity and specificity for detecting both IgG and IgM by ELISA in suspected scrub typhus patients [13], thus making it a suitable candidate for replacing the density gradient-purified, rickettsia-derived, whole-cell antigen. The 56-kDa is immunodominant. Immunization of its recombinant protein was protective in mice models and conferred both humoral and cellular immunity [25]. The recombinant 56-kDa protein also demonstrated its advantage in dot-ELISA and rapid diagnostic test [26,27]. In those reports, recombinant 56-kDa proteins derived from *O. tsutsugamushi* Karp, Kato, Gilliam, and local serotypes were applied. In assays using a single serotype, the Karp-derived antigen has been widely used [13,26], potentially due to its widespread in endemic areas [10]. A combination of multiple serotypes-derived proteins (either 56-kDa proteins from different serotypes or 56-kDa and other proteins) was evaluated to improve sensitivity and specificity [27,28].

Herein, the recombinant 56-kDa type-specific antigen (TSA56) derived from the *O. tsutsugamushi* Karp serotype was cloned, expressed, and tested for its ability to reveal immune status during scrub typhus infection using indirect IgM and IgG ELISAs. The developed ELISA assays were tested with scrub typhus patient sera and other febrile illnesses, i.e., murine typhus, melioidosis, leptospirosis, and dengue from Thailand.

## 2. Materials and Methods

### 2.1. Genomic DNA

Genomic DNA used in the cloning experiments was extracted from *O. tsutsugamushi* Karp serotype and cultured in a Vero cell by a commercial genomic DNA extraction kit. (TIANamp bacteria DNA kit, Tiangen Biotech, Beijing, China). Bacterial cell lysis was cultivated and prepared in a biosafety level 3 facility.

### 2.2. Construction of Recombinant Plasmid

The PCR product derived from the gene encoding 56-kDa type-specific antigen (TSA56) of *O. tsutsugamushi* was cloned into a pRSET-B vector (Invitrogen Ltd., Paisley, UK) composed of the 6× Histidine tag at the 5′ end of the inserted DNA. The oligo primer pairs were designed to incorporate *Xho*I at the 5′ end and *Eco*RI at the 3′ end (detailed sequences shown in Table 1). The PCR product and plasmid vector were double-digested with those two enzymes and ligated. The obtained recombinant plasmid was transformed into an *E. coli* DH5α host strain. DNA sequencing verified positive clones’ DNA insert, and the recombinant plasmid was then transformed into the *E. coli* BL-21(DE3) strain containing pLysS with chloramphenicol resistance (Stratagene, Santa Clara, CA, USA; Cat. #200133).

### 2.3. Expression and Purification of 56-kDa Recombinant Protein

The recombinant protein was expressed by adding IPTG (isopropyl-D-thiogalactopyranoside) to obtain a final concentration of 1 mM during the late log phase culture. After three hours of induction, the bacterial cell pellet was collected and gently lysed in BugBuster^®^ Master Mix buffer (Novagen, Madison, WI, USA). The production of the recombinant protein, either in solubilized or insolubilized form, was confirmed by SDS-PAGE and by the His-tag marker of the fractionated protein. The His-tagged proteins were purified using Protino^®^ Ni-TED Resin (MACHEREY-NAGEL, Bethlehem, PA, USA) gravity-flow column chromatography under either native or denaturing conditions. After binding the His-tagged fusion proteins to the nickel affinity column, the protein was eluted by a gradient concentration of imidazole ranging from 0 to 0.5 M. The purified protein fraction was collected, subjected to dialysis in 0.01 M PBS pH 7.2 overnight, and then concentrated by freeze-dried lyophilization.

### 2.4. Serum Samples

The study protocol involving human specimens was approved by the Ethics Committee of the Faculty of Tropical Medicine, Mahidol University (approval no. MUTM 2021-038-01). The main target study group comprised scrub typhus patients, of which single sera was obtained and verified positive by IFA assay (92 samples). The comparative study included the following groups: healthy normal people (31 samples), murine typhus patients (25 samples), leptospirosis patients (42 samples), dengue patients (35 samples), and melioidosis patients (36 samples).

### 2.5. Indirect ELISA

Each well of an ELISA plate was coated with 100 µL per well of purified recombinant protein diluted in carbonate–bicarbonate buffer pH 9.6, based on the optimal concentration of protein Ag. Recombinant 56-kDa proteins of varying concentrations ranging from 100 to 3 µg/mL were coated on the plate to determine the optimized concentration of protein Ag. The coating plates were then incubated at 4 °C in a humid chamber overnight. Unbound antigen was removed by washing with PBS-tween four times, and all wells were blocked with 200 µL of 1% BSA (bovine serum albumin) at 37 °C for one hour. Excess BSA was washed off, and 100 µL of the serum sample diluted with diluent (0.2% gelatin + 0.2% BSA in PBS) was added. Blank wells containing diluent instead of serum were included in the ELISA plate. The antigen–antibody reaction was allowed to take place at 37 °C for one hour. The washing step was performed before adding 100 µL of goat antihuman IgG or IgM conjugated to horseradish peroxidase (Southern Biotech, Birmingham, AL, USA), 1:4000 dilution, for one hour. After thorough washing, 100 µL of ABTS (2,2′-azinobis [3-ethylbenzothiazoline-6-sulfonic acid] diammonium salt substrate) (KPL, Gaithersburg, USA) was added, and the wells were incubated for 20 min. The reaction was stopped with 100 µL of 1% SDS. The absorbance value was then measured at a wavelength of 405 nm (OD_405_) by an ELISA reader (Tecan Group Ltd., Männedorf, Switzerland). Each ELISA experiment included one positive serum and one negative serum in each plate. These control optical densities (ODs) had minimal variation per experiment, not more than ± 0.1, to validate other experimental ODs.

### 2.6. Data Analysis

IgM and IgG antibodies to the recombinant TSA56 protein were determined among scrub typhus patients and other study groups. Serological titration data were converted into logarithm to the base 2 (LN), as titrations of antiserum are commonly made by serial two-fold dilutions [29]. The mean and (+/−) standard deviation (SD) derived from the normalized ELISA data (OD_405_ and LN titer) were calculated. Comparisons of means by ANOVA and *t*-test and analysis of receiver operating characteristic (ROC) were performed in GraphPad Prism version 7. The information obtained by comparing the ELISA results and the scrub typhus status was conventionally summarized in a two-by-two table. Sensitivity, specificity, positive predictive value (PPV), and negative predictive value (NPV) were calculated as described elsewhere [30].

## 3. Results

### 3.1. DNA Cloning and Protein Expression

The 56-kDa type-specific antigen (TSA56) gene of the locus tag UT176_00941, located on the *O. tsutsugamushi* isolate UT176 chromosome I (Sequence ID: NZ_LS398547.1), was the reference gene for the primer design. The forward primer was designed after the signal peptide and expanded to the conserved region on the 5′ end of the 56-kDa TSA gene. The partial DNA sequence of the TSA56 gene was cloned into the pRSET-B vector, and the positive clone K13 was obtained. Its inserted DNA sequence was aligned well with other TSA genes from the known strains of *O. tsutsugamushi* (Figure S1). The size of the K13 insert was 1023 bp, encoded for 341 amino acids, of which its predicted molecular weight was 36.51 kDa. A sequence of the DNA insert was submitted to GenBank database and received an accession no. OP681316.

### 3.2. Recombinant Protein Expression and Purification

**Figure 1:**
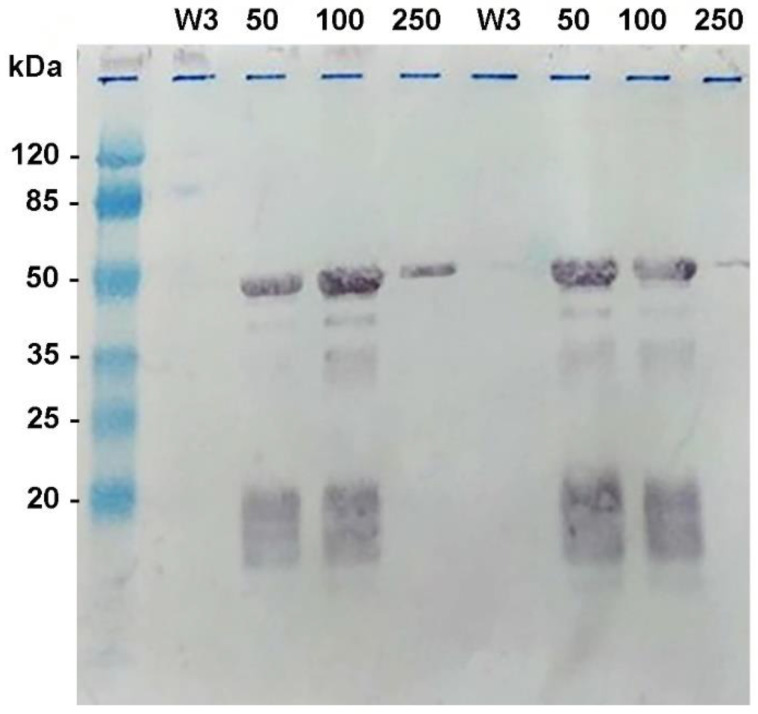
Western blot analysis of the histidine-tagged recombinant TSA56 protein expressed from the K13 clone. The eluted protein was shown during nickel affinity column purification. After the histidine-tagged protein was bound to nickel, it was washed (lanes W3) and then eluted in 5 mL fractions with 50 mM, 100 mM, and 250 mM imidazole (lanes 50, 100, and 250). Western blot of the recombinant protein from two expression experiments is shown.

Sequencing analysis revealed the DNA insert from *Xho*I to *Eco*RI site to be 1073 bp, which was predicted for an open reading frame encoding 341 amino acids of molecular weight (MW) 36.51 kDa. The log phase of the recombinant clone was induced by 1 mM IPTG for 3 h; the cell pellet was subjected to protein extraction by gentle lysis with a commercial lysis solution (BugBuster^®^). The protein expressed by the K13 clone mainly resided at the inclusion body and required 8 M urea for solubilization. The purification procedure based on an affinity of the histidine fusion protein to the nickel column needed to be performed in a denatured solution with 8 M urea. The nickel column was incubated with the histidine fusion protein, and after washing unbound protein, which was shown in lane W3, an attempt to elute the bound protein was performed with 50 mM, 100 mM, and 250 mM imidazole, as demonstrated in Figure 1. By Western blot analysis of the expressed recombinant protein, the higher MW of approximately 48 kDa was detected, indicating that some part of DNA vector was transcribed and attached to this protein. The purified protein from a few batches of cell pellet were prepared, and approximately 23 mg of protein was obtained.

### 3.3. Optimization of Recombinant TSA56 Antigen Concentration in ELISA Assay

The ELISA plate was coated with varying concentrations of the recombinant TSA56 protein: 100, 50, 25, 12.5, 6, and 3 µg/mL. Serum from one scrub typhus patient was subjected to two-fold dilution, starting from one in 400, 800, 1600, 3200, 6400, 12,800, and 25,600. The IgM and IgG conjugates were used at dilution 1:4000, and the OD_405_ cut-off was read at 0.3. For IgM ELISA, as shown in Table 2, positive results can be read for coated antigen concentration as low as 3 µg/mL at the end point of serum dilution 1:3200. When the IgG ELISA determined the same control patient serum, the endpoint of positive serum dilution was read at 1:400 dilutions, with coated antigen concentration as low as 12.5 µg/mL (Table 3). Therefore, a protein concentration of 10 µg/mL was used in plate coating for economization in further experiments.

### 3.4. Evaluation of TSA56 Recombinant Protein for Scrub Typhus Diagnosis by Indirect ELISA

**Figure 2:**
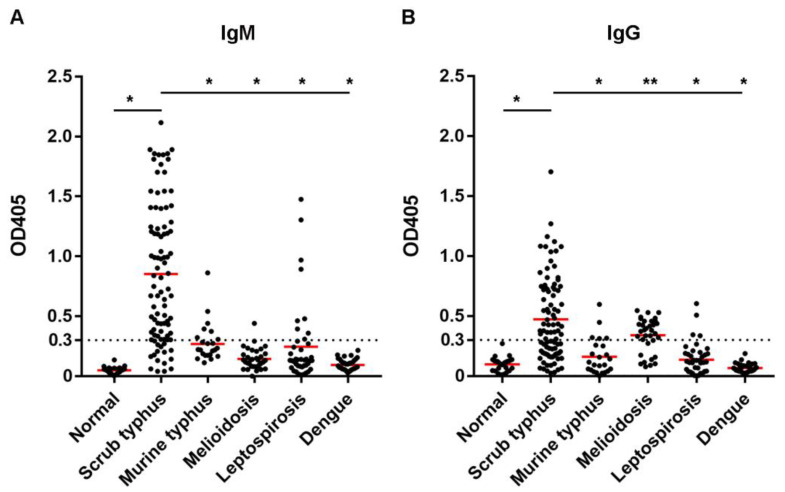
Indirect TSA56 ELISA results. IgM (**A**) and IgG (**B**) ELISAs were performed using the TSA56 recombinant protein as a coating antigen. The sera dilution was at 1:400. OD_405_ at 0.3 was set as an assay cut-off. Red lines represent mean OD. *, *p* < 0.0001; **, *p* = 0.0029.

An antibody against the TSA56 recombinant protein was revealed among scrub typhus patients by indirect IgM and IgG ELISAs compared to the normal group. The patient sera were scrub typhus-positive by IFA based on native antigens from cells infected with *O. tsutsugamushi* of three serotypes (Karp, Gilliam, and Kato). Figure 2 shows results when the sera were tested at dilution 1:400. In IgM ELISA, the mean ± SD of OD_405_ was observed as 0.049 ± 0.029 for normal sera and 0.852 ± 0.571 for scrub typhus-positive sera. In IgG ELISA, the mean ± SD of OD_405_ was observed as 0.097 ± 0.057 for normal sera and 0.473 ± 0.343 for typhus-positive sera. The mean OD_405_ of scrub typhus sera was significantly higher than those of normal sera in both IgM and IgG assays (*p* < 0.0001). Receiver operating characteristic (ROC) analysis was performed to determine the assay sensitivity and specificity at different OD_405_ cut-offs (Table 4). At the cut-off OD_405_ 0.3, as was used in the assay optimization, 80.4% sensitivity and 100% specificity were obtained for IgM assay with the area under the curve (AUC) 0.978, whereas 58.7% sensitivity and 100% specificity were obtained for IgG assay with AUC 0.903. Table 5 shows a number of serum samples defined as scrub typhus-positive using different assay cut-offs.

The TSA56 indirect ELISA was also performed with sera collected from patients with other febrile illnesses to observe its cross-reactivity with other bacterial and viral infections, i.e., murine typhus, melioidosis, leptospirosis, and dengue (Figure 2). At serum dilution 1:400 and OD_405_ cut-off at 0.3, cross-reactivity could be observed when the ELISAs were conducted using bacterial infections but not dengue patient sera. In the IgM assay, 29.2% of murine typhus, 2.9% of melioidosis, and 21.4% of leptospirosis sera showed positive results. In IgG assay, up to 70.6% of sera from melioidosis patients showed IgG antibody positive, whereas 20.8% of murine typhus- and 9.5% of leptospirosis-positive sera showed positive results. For IgM ELISA, the means ± SD of OD_405_ were 0.269 ± 0.171 for murine typhus, 0.144 ± 0.086 for melioidosis, 0.245 ± 0.331 for leptospirosis, and 0.094 ± 0.050 for dengue sera. For IgG ELISA, the means ± SD of OD_405_ were 0.162 ± 0.151 for murine typhus, 0.341 ± 0.143 for melioidosis, 0.137 ± 0.131 for leptospirosis, and 0.068 ± 0.036 for dengue sera. Means OD_405_ of all other febrile sera were significantly lower than scrub typhus when tested by both IgM and IgG ELISA (*p* < 0.0001 for all conditions, except *p* = 0.0029 for melioidosis sera in IgG ELISA). It was shown by ROC analysis that the TSA56 IgM ELISA sensitivity could be increased from 80.4% to 89.1% if the OD_405_ cut-off was set at 0.2 (Table 4). Based on the probability of cross-reaction, OD_405_ at 0.3 was retained as the cut-off OD for both TSA56 IgM and IgG ELISAs.

Antibody titer response to the TSA56 recombinant protein was further revealed among scrub typhus patients compared to the titer from normal and the other disease groups. The titers were transformed to natural logarithm (LN) prior to analysis [29], i.e., 5.3, 5.99, 6.68, 7.38, 8.07, 8.76, 9.46, and 10.15 for titers 200, 400, 800, 1600, 3200, 6400, 12,800, and 25,600, respectively. An antibody titer of less than 200 was assigned as 1. The LN of 1 was 0. Samples with an antibody titer more than 25,600 were assigned as titer 25,600, and their LN was 10.15. As shown in Table 6, the mean LN IgM titer of the normal group was 2.39 ± 2.68, which was lower than the mean LN IgM titer of 6.80 ± 2.61 of the scrub typhus group (*p* < 0.0001). The mean LN IgG titer of the normal group was 0.51 ± 1.59, which was significantly lower than the mean LN IgG titer of 5.18 ± 2.81 of the scrub typhus group (*p* < 0.0001). The means of LN IgM and LN IgG titers of scrub typhus patients were also significantly higher than the mean titers from other febrile patients. However, mean LN IgM titer of 3.89 ± 3.08 (median 5.30) and LN IgG titer of 3.53 ± 2.53 (median 5.30) were observed when the ELISAs were tested with murine typhus and melioidosis sera, respectively (Table 6). Although a significant difference was found (*p* = 0.0001 and *p* = 0.002, respectively), it indicates that a cut-off titer should be carefully set to decrease cross-reactivity with the other bacterial infections.

**Figure 3:**
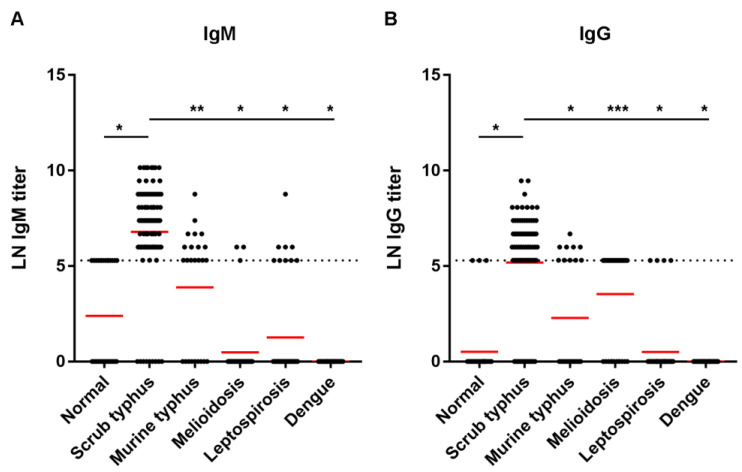
LN IgM titers (**A**) and LN IgG titers (**B**) to the recombinant TSA56 protein assayed by indirect ELISA among normal, scrub typhus, and the other disease groups. The dashed line represents LN titer 5.3 (titer 200). Red lines represent means of LN titers. *, *p* < 0.0001; **, *p* = 0.0001; ***, *p* = 0.002.

The scatter plots of LN IgM titer (Figure 3A) and LN IgG titer (Figure 3B) among scrub typhus patients, normal patients, and those with other diseases were created. LN IgM and IgG titers up to 5.3 (titer 200) were detected in the normal group. Therefore, the cut-off titer for positive scrub typhus was set at an antibody titer equal to or higher than 400 (LN = 5.99) in either IgM or IgG ELISAs. Using such criteria, the sensitivity of the TSA56 IgM ELISA was 87.0%, and the specificity was 100% (Table 7). The sensitivity of IgG ELISA was 59.8%, and the specificity was 100%. When IgM and IgG ELISA results were combined, an increase in sensitivity to 95.7% was obtained. However, cross-reactivity was still observed with some of the sera infected with the other bacterial infections (Figure 3 and Table 6).

Among the scrub typhus group, 33 patients showed IgG titer of 200 (LN = 5.3) or less, while their IgM titers ranged from 400 to 25,600 (LN = 5.99 to 10.15), indicating the acute stage of scrub typhus infection. In contrast, there were eight patients whose IgM titers were equal to or less than 200, while their IgG titers ranged from 400 to 1600. This could indicate a convalescent stage of infection.

## 4. Discussion

This study selected the 56-kDa type-specific antigen (TSA) of the *O. tsutsugamushi* Karp serotype as a target for recombinant protein production and development of IgM and IgG ELISA assays. 56-kDa TSA is an immunogenic protein recognized by scrub typhus patient sera [13]. Immunizations of the recombinant 56-kDa proteins demonstrated protection against homologous and heterologous *O. tsutsugamushi* strains [25,31]. Its uses as a target for both serological and molecular diagnosis were widely described [16] and in vaccine development [32]. *Orientia tsutsugamushi* was initially characterized into three prototypic serotypes, Karp, Gilliam, and Kato [10]. Later, more serotypes were identified, including various new strains from Thailand and Japan. DNA sequence analysis, especially that based on 56-kDa TSA gene, was used for bacterial genotype identification by phylogenetics. Numbers of *O. tsutsugamushi* genotypes, either related or separated from the prototypes Karp, Gilliam, and Kato, were identified. Among them, Karp and Karp closely related serotypes commonly cause infections in the areas of endemicity [10]. In a study using 56-kDa TSA-specific PCR, the Karp serotype was detected in 97% of the scrub typhus-positive patient samples from Thailand, with 3% of the samples positive for the Kato serotype and none of them positive for the Gilliam serotype [33]. However, Gilliam and the other antigenically distinct Thai strains were reported from humans, mites, and animals in Thailand as a minority [10,34]. 56-kDa TSA gene has some diversities among *O. tsutsugamushi* strains. Comparison of the 56-kDa TSA nucleotide sequences of Thai *O. tsutsugamushi* isolates, three prototype strains, and isolates from other Asian countries revealed a range of 78–96% nucleotide sequence identity [34]. The variation observed at the gene level could contribute to the antigenicity of proteins. Nevertheless, cross-reactivity was observed when various 56-kDa recombinant protein antigens were reacted with scrub typhus serum samples [35]. Based on the strain distribution information, the Karp antigen is the best choice for a single-antigen immune assay. To increase the power of detection, it was suggested by data from Thailand that, in addition to Karp, a pool of antigens for serological diagnosis may also include Gilliam, TA716, TA763, and Kato strain antigens [34]. Expression and comparison of recombinant 56-kDa proteins derived from different bacterial isolates, as well as a protein conformational assessment and removal of a co-purified vector part, are other approaches that can be attempted for improving the recombinant protein performance.

One factor that determines the diagnostic assay accuracy is a cut-off setting to discriminate between positive and negative. When the cut-off is changed, a positive rate and negative rate, as well as the sensitivity and specificity of the test, are changed. Receiver operating characteristic (ROC) analysis is widely used to define an appropriate cut-off [36]. Using a set of data from normal and disease groups, the ROC curve shows a true positive rate (sensitivity) against a false positive rate (1—specificity), hence depicting a trade-off between a test sensitivity and specificity for each cut-off value [36,37]. Sensitivity and specificity are altered oppositely, i.e., if the test cut-off was decreased to increase sensitivity, specificity will be reduced, and vice versa. To choose an appropriate cut-off, the most common criteria are selecting the point on the ROC curve where the sensitivity and specificity of the test are equal [36]. Based upon the ROC analysis, it could be seen that the cut-off OD_405_ 0.2 or lower may be selected for a compromised sensitivity and specificity for both IgM and IgG assays in our study. However, at OD_405_ 0.2, a high cross-reactivity rate could be obtained when the assays were tested against other bacterial infection sera (Figure 2). Therefore, OD_405_ 0.3 was used as a positive cut-off in both IgM and IgG assays. There were also reports showing cross-reactivity of the scrub typhus immunoassays with sera from patients with murine typhus (16% with IgM immunochromatographic test) and leptospirosis (20% with recombinant 56-kDa IgG ELISA) [38,39]. It was suggested that the cut-off value is not universal and should be defined for each region and each disease conditions [30,36]. For example, in the areas where other febrile illnesses that can show similar clinical manifestations as scrub typhus is endemic, a specific diagnostic test is required to differentiate the diseases. Therefore, proper treatment can be given to decrease time and cost.

A cut-off titer is also useful in interpreting disease status, especially in the endemic area where the population may have pre-existing immunity. Using IFA IgM as an example, cut-off titers of ≥400 in an admission sample or a ≥four-fold rise to titer ≥200 in a convalescent-phase selection were first proposed in 1983 and have been conventionally used for the scrub typhus diagnosis. However, a high positivity rate was suspected with these cut-offs [40]. In 2011, the scrub typhus infection criteria (STIC) were applied using a combination of culture, PCR assays, and IFA IgM for defining the scrub typhus status [18]. STIC uses cut-off titers of either ≥12,800 in an admission sample or a ≥four-fold rise in a convalescent-phase sample. The criteria have been used as a comparator to evaluate the accuracy of alternative diagnostic tests [17]. By using an unbiased Bayesian approach to the data set from Thailand, it was later suggested that optimal cut-off titers for IFA IgM as either ≥3200 in an admission sample or as a ≥four-fold rise to ≥3200 in a convalescent-phase sample could improve the assay sensitivity and specificity [40]. Assays, including pooled whole cell ELISA, recombinant 56-kDa ELISA, and dipstick tests, were evaluated for their sensitivity and specificity at different titer cut-offs [39]. It was shown that the recombinant IgM ELISA gave sensitivity and specificity of 93% and 94%, respectively, at cut-off titer 400, and IgG ELISA gave sensitivity and specificity of 97% and 92%, respectively, at cut-off titer 1600 [39]. Due to its performance and use of a standardized antigen, the recombinant ELISA is considered suitable in a diagnostic laboratory to replace IFA. In our study, we retrieved up to 100% specificity in both IgM and IgG ELISAs, and 87% sensitivity for IgM ELISA, at the cut-off titer 400. The results suggested that the IgM ELISA could be used for diagnosis. Low sensitivity of 60% of IgG ELISA indicates a need for further improvement and evaluation. Nevertheless, the test performance increased to 96% sensitivity when combined with IgM and IgG results.

Although various molecular and serological assays have been developed and evaluated for scrub typhus diagnosis, no assay showed a sufficiently influential performance to be used alone. Each assay has its own advantage. They are affected by different factors making them imperfect. Molecular assays, including real-time PCR, nested PCR, multiplex PCR, and LAMP, provided sensitivity and specificity in range of 20.6–86% and 70.5–100%, respectively [16]. The nucleic acid-based molecular assays are only beneficial for diagnosis at the early stage of infection when the bacteria agent is present in the patient specimens. Antibody-based serological assays, on the other hand, can be used to diagnose the disease in various stages. IgM antibody is detected at 4–7 days postinfection and was shown to maintain up to 12 months in scrub typhus patients [41]. IgG antibody increases after that and maintains that level for months or years. IgG was reported in scrub typhus patients up to more than 3 years postinfection [41]. Therefore, specific IgM detection is recommended for the diagnosis of primary or acute infection. IgG-based methods are not able to differentiate between a recent and past infection but useful in an epidemiological study of the disease prevalence [16]. In previous studies, 65.7–100% sensitivity and 72.5–95.5% specificity were reported for IgM ELISA; 66.2–91% sensitivity and 75–86.1% specificity were reported for IgG ELISA. IgM IFA showed 70–100% sensitivity and 83.8–93.5% specificity [16,42,43]. Our IgM ELISA is not inferior to the other assays in both sensitivity and specificity. The use of recombinant protein also provides an advantage for antigen standardization and removes the issue of BSL3 facility requirement. Therefore, it deserves further improvement and evaluation to use in the scrub typhus diagnosis, either as a single test or in combine with other assays. The recombinant 56-kDa protein itself can also be used in a development of the other assays such as a lateral flow immunochromatographic assay.

The limitation of our study is based mainly on identifying the test samples. IFA for scrub typhus was performed in only scrub typhus patients, not on other comparative groups, and the status of IgM and IgG was unknown. Information on how long the patients had been symptomatic, which could influence the serological reactivity, was also unavailable. Therefore, determining both IgG and IgM could increase the diagnostic performance, as in some cases, IgM titer was high, while no or low titer of IgG was detected. Since the assay showed a low sensitivity for IgG detection, in case of samples diagnosed later during the clinical course in which IgM is absent, combined IgM/IgG may not provide higher performance compared to IgG alone. The cut-off titer 400 was set merely based on the baseline titer observed in the normal group, which could be more accurate if the scrub typhus status of the comparative group was defined. Due to the limitation, a conclusion could not be made whether the cross-reactivity with murine typhus, melioidosis, and leptospirosis occurred because of a nonspecific reactivity of the recombinant protein with factors in those bacterially infected sera, a scrub typhus coinfection or pre-existing antibodies. Concerning the diagnosis cut-off, it can be recommended that a revision be performed from time to time using standardized and unbiased methods, together with a set of representative data from endemic and nonendemic areas, to justify an appropriate cut-off for a particular disease diagnosis. Developing a new test with improved performance is also essential for evaluation.

## 5. Conclusions

We produced the recombinant 56-kDa TSA antigen of scrub typhus Karp serotype and used it in indirect IgM and IgG ELISAs. High sensitivity and specificity, 87% and 100%, respectively, were obtained for IgM ELISA at cut-off titer 400. The sensitivity was increased when combined IgM and IgG assays were considered. The recombinant protein showed potential for further evaluation and development in scrub typhus diagnosis.

## Figures and Tables

**Table 1:** Primers designed for amplifying the *O. tsutsugamushi* 56-kDa type-specific antigen by polymerase chain reaction (PCR).

Protein (Locus Tag)	Strand	Primer Sequence (5′-3′)	Restriction Enzyme Site	Size of PCR Product (bp)
UT176_00941	plus	GGGACTCGAGAGGATTAGAGTGTGGTC	*Xho*I	1058
	minus	ACGCTGGAATTCAACAAGATCTCTATAT	*Eco*RI	

Underline indicates the recognition site of *Xho*I or *Eco*RI.

**Table 2:** OD_405_ from the ELISA plate, coated with the calibrating recombinant protein, reacting with scrub typhus positive serum, using IgM conjugate dilution of 1:4000.

Protein Conc. (μg/mL)	Serum Titer							
	400	800	1600	3200	6400	12,800	25,600	NC
100	1.530	1.282	0.734	**0.482**	0.353	0.196	0.107	0
50	2.058	1.640	0.807	**0.426**	0.178	0.077	0.037	0
25	1.916	1.688	0.903	**0.587**	0.326	0.201	0.111	0.002
12.5	1.717	1.482	0.851	**0.560**	0.345	0.208	0.110	0.006
6	1.502	1.206	0.728	**0.485**	0.320	0.187	0.100	0.011
3	1.267	1.148	0.646	**0.407**	0.270	0.165	0.092	0

NC, negative control; Bold numbers represent the end-point reading.

**Table 3:** OD_405_ from the ELISA plate, coated with the calibrating recombinant protein, reacting with scrub typhus positive serum, using IgG conjugate dilution of 1:4000.

Protein Conc. (μg/mL)	Serum Titer							
	400	800	1600	3200	6400	12,800	25,600	NC
100	**0.399**	0.267	0.156	0.079	0.041	0.032	0.007	0
50	**0.408**	0.247	0.220	0.075	0.029	0	0.011	0
25	**0.413**	0.255	0.087	0.044	0.027	0.012	0.044	0.085
12.5	**0.328**	0.221	0.071	0.047	0.009	0.009	0.109	0
6	0.297	0.163	0.101	0.052	0.006	0.008	0.073	0.079
3	0.223	0.126	0.071	0.009	0.003	0.038	0	0.005

NC, negative control; Bold numbers represent the end-point reading.

**Table 4:** Sensitivity and specificity of TSA56 indirect ELISA at different OD_405_ cut-offs.

OD_405_ Cut-Off	IgM		IgG	
	% Sensitivity	% Specificity	% Sensitivity	% Specificity
0.5	60.9 (50.7–70.2)	100 (89.0–100)	40.2 (30.8–50.4)	100 (89.0–100)
0.4	69.6 (59.5–78.0)	100 (89.0–100)	47.8 (37.9–57.9)	100 (89.0–100)
0.3	80.4 (71.2–87.3)	100 (89.0–100)	58.7 (48.5–68.2)	100 (89.0–100)
0.2	89.1 (81.1–94.0)	100 (89.0–100)	72.8 (63.0–80.9)	96.8 (83.8–99.8)
0.1	94.6 (87.9–97.7)	96.8 (83.8–99.8)	90.2 (82.4–94.8)	45.2 (29.2–62.2)

The numbers in parenthesis indicate 95% CI.

**Table 5:** Numbers of scrub typhus positive serum samples analyzing by different OD_405_ cut-offs.

OD_405_ Cut-Off	IgM		IgG	
	Normal (n = 31)	ST Sera (n = 92)	Normal (n = 31)	ST Sera (n = 92)
0.5	0	56 (60.7%)	0	37 (34.0%)
0.4	0	64 (69.6%)	0	44 (47.8%)
0.3	0	76 (82.6%)	0	54 (58.7%)
0.2	0	83 (90.2%)	1 (3.2%)	67 (72.8%)
0.1	1 (3.2%)	88 (95.7%)	17 (54.8%)	83 (90.2%)

**Table 6:** Comparison of means and medians of LN value of the endpoint antibody titer and cross-reactivity for IgG and IgM ELISAs among studied groups.

	Subject Groups	Mean ± SD	Median	No. Positive for ST ELISA (%)
LN IgM	Scrub typhus	6.80 ± 2.61	7.38	80/92 (87.0%)
	Normal	2.39 ± 2.68 *	0	0/31
	Murine typhus	3.89 ± 3.08 **	5.30	9/25 (36%)
	Melioidosis	0.48 ± 1.62 *	0	2/36 (5.6%)
	Leptospirosis	1.27 ± 2.51 *	0	4/42 (9.5%)
	Dengue	0 *	0	0/35
LN IgG	Scrub typhus	5.18 ± 2.81	5.99	55/92 (59.8%)
	Normal	0.51 ± 1.59 *	0	0/31
	Murine typhus	2.29 ± 2.87 *	0	5/25 (20%)
	Melioidosis	3.53 ± 2.53 ***	5.30	0/36
	Leptospirosis	0.50 ± 1.57 *	0	0/42
	Dengue	0 *	0	0/35

ST, scrub typhus; The mean LN titer is significantly different from that of the scrub typhus group at *p* < 0.0001 (*), *p* = 0.0001 (**), and *p* = 0.002 (***).

**Table 7:** Numbers of scrub typhus antibody-positive and negative sera tested by IgG and IgM ELISAs using titer cut-off at 400.

	**Scrub Typhus Status**				
**IgM ELISA**	**No. of Positive**	**No. of Negative**	**Total**		**(%)**
No. of positive	80	0	80	Sensitivity	87.0
No. of negative	12	31	43	Specificity	100
Total	92	31	123	PPV	100
				NPV	72.1
	**Scrub typhus status**				
**IgG ELISA**	**No. of positive**	**No. of negative**	**Total**		**(%)**
No. of positive	55	0	55	Sensitivity	59.8
No. of negative	37	31	68	Specificity	100
Total	92	31	123	PPV	100
				NPV	45.6
	**Scrub typhus status**				
**IgM or IgG ELISA**	**No. of positive**	**No. of negative**	**Total**		**(%)**
No. of positive	88	0	88	Sensitivity	95.7
No. of negative	4	31	35	Specificity	100
Total	92	31	123	PPV	100
				NPV	88.6

PPV, positive predictive value; NPV, negative predictive value.

## Data Availability

Data are contained within the article and Supplemental Materials. A nucleotide sequence of the recombinant DNA was submitted to GenBank (https://www.ncbi.nlm.nih.gov/genbank/) and received an accession no. OP681316 (accessed on 1 October 2022).

## Supplementary Materials

The following supporting information can be downloaded at: https://www.mdpi.com/article/10.3390/tropicalmed8010010/s1, Figure S1: Multiple DNA alignment of 56-kDa TSA (type-specific antigen) gene among varied strains of *O. tsutsugamushi*.
