# Supplementary material for: A Use of 56-kDa Recombinant Protein of Orientia tsutsugamushi Karp Serotype in Serodiagnosis of Scrub Typhus by Enzyme-Linked Immunosorbent Assay in Thais
Source: Trop Med Infect Dis. 2022 Dec 23;8(1):10. doi: 10.3390/tropicalmed8010010 (PMC9865064; doi:10.3390/tropicalmed8010010)
Supplement: Supplementary file 1 [file tropicalmed-08-00010-s001.zip › tropicalmed-2027806-supplementary.pdf]

```
#EF213078.1_isolte_UT76_strain_Karp           --- --- --- --- --- --T GAA AAA AAT TAT GTT AAT TGC TAG TGC AAT GTC TGC GTT GTC  [ 600]
#EF213086.1_isolate_UT150_strain_Karp         --- --- --- --- --- --. ... ... ... ... ... ... ... ... ... ... ... ... ... ...  [ 600]
#EF213096.1_isolate_UT125_strain_Gilliam      --- --- --- --- --- --. ... ... ... ... ... ... ... ... ... ... ... ... ... ...  [ 600]
#EF213091.1_isolate_UT144_strain_Gillium      --- --- --- --- --- --. ... ... ... ... ... ... ... ... ... ... ... ... ... ...  [ 600]
#M63382.1_strain_Kato                         TTT TAA GGA GAT TAG AA. ... ... ... ... ... ... ... ... ... ... ... ... A.. ...  [ 600]
#EF213078.1_isolate_FPW1038_strain_TA716      --- --- --- --- --- --. ... ... ... ... ... ... ... ... ... ... ... ... ... ...  [ 600]
#EF213095.1_isolate_UT302_strainTA763         --- --- --- --- --- --. ... ... ... ... ... ... ... ... ... ... ... ... ... ...  [ 600]
#K13_insert                                   --- --- --- --- --- --- --- --- --- --- --- --- --- --- --- --- --- --- --- ---  [ 600]

#EF213078.1_isolte_UT76_strain_Karp           GTT GCC GTT TTC TGC TAG TGC GAT AGA ATT GGG GGA TGA AGG AGG ATT AGA GTG TGG TCC  [ 660]
#EF213086.1_isolate_UT150_strain_Karp         ... ... ... ... ... ... ... ... ... ... ... ... ... ... ... ... ... ... ... ...  [ 660]
#EF213096.1_isolate_UT125_strain_Gilliam      ... ... ... ... A.. ... ... ... ... ... ... ... ... ... ... ... ... ... ... ...  [ 660]
#EF213091.1_isolate_UT144_strain_Gillium      ... ... ... ... A.. ... ... ... ... ... ... ... ... ... ... ... ... ... ... ...  [ 660]
#M63382.1_strain_Kato                         A.. ... ... ... A.. ... ... ... ... ... ... ... ... ... ... ... ... ... ... ...  [ 660]
#EF213078.1_isolate_FPW1038_strain_TA716      ... ... ... ... ... ... ... ... ... ... ... ... ... ... ... ... ... ... ... ...  [ 660]
#EF213095.1_isolate_UT302_strainTA763         ... ... ... ... A.. ... ... ... ... ... ... ... ... ... ... ... ... ... ... ...  [ 660]
#K13_insert                                   --- --- --- --- --- --- --- --- --- --- --- --- --- -A. ... ... ... ... ... ...  [ 660]

#EF213078.1_isolte_UT76_strain_Karp           TTA TGC TAA AGT TGG AGT CGT TGG AGG AAT GAT TAC TGG CGT AGA ATC TAT TCG CTT GGA  [ 720]
#EF213086.1_isolate_UT150_strain_Karp         ... ... ... ... ... ... ... ... ... ... ... ... ... ... ... ... ... ... ... ...  [ 720]
#EF213096.1_isolate_UT125_strain_Gilliam      ... ... ... ... ... ... ... ... ... ... T.. ... ... ... ... ... ..C ... ... ...  [ 720]
#EF213091.1_isolate_UT144_strain_Gillium      ... ... ... ... ... ... ... ... ... ... T.. ... ... ... ... ... ..C ... ... ...  [ 720]
#M63382.1_strain_Kato                         ... ... ... ... ... ... ... ... ... ... ... ... ... ... ... ... ..C ... ... ...  [ 720]
#EF213078.1_isolate_FPW1038_strain_TA716      ... ... ... ... ... ... ... ... ... ... ... ... ... ... ... ... ... ... ... ...  [ 720]
#EF213095.1_isolate_UT302_strainTA763         ... C.G ... ... ... .A. T.. ... ... ... ... ... ... T.. ... ... ..C ..A ... ...  [ 720]
#K13_insert                                   ... ... ... ... ... ... ... ... ... ... ... ... ... ... ... ... ... ... ... ...  [ 720]

#EF213078.1_isolte_UT76_strain_Karp           TCC AGC TGA TGC TGA TGG CAA AAA ACA CTT GCC ATT AAT AAC CTC GAT GCC ATT TGG TGG  [ 780]
#EF213086.1_isolate_UT150_strain_Karp         ... ... ... ... ... ... ... ... ... ... ... ... ... ... ... ... ... ... ... ...  [ 780]
#EF213096.1_isolate_UT125_strain_Gilliam      ... ... ... .A. .A. ... ... ... .T. ... ... ... ..C ... ... ... ... ... ... ...  [ 780]
#EF213091.1_isolate_UT144_strain_Gillium      ... ... ... .A. .A. ... ... ... .T. ... ... ... ..C ... ... ... ... ... ... ...  [ 780]
#M63382.1_strain_Kato                         ... ... ... ... ..G ... ... ... ... A.. ... ... ..C ... ... ... ... ... ... ...  [ 780]
#EF213078.1_isolate_FPW1038_strain_TA716      ... ... ... ... ... ... ... ... ... ... ... ... ... ... ... ... ... ... ... ...  [ 780]
#EF213095.1_isolate_UT302_strainTA763         .T. ... ... ... ..G A.. ... ... ... ... ... ... ..C ... ... ... ... ... ... ...  [ 780]
#K13_insert                                   ... ... ... ... ... ... ... ... ... ... ... ... ... ... ... ... ... ... ... ...  [ 780]

#EF213078.1_isolte_UT76_strain_Karp           TAC ATT AGC TGC AGG TAT GAC AAT TGC TCA AGG TTT TAG AGC AGA GCT GGG GGT TAT GTA  [ 840]
#EF213086.1_isolate_UT150_strain_Karp         ... ... ... ... ... ... ... ... ... ... ... ... ... ... ... ... ... ... ... ...  [ 840]
#EF213096.1_isolate_UT125_strain_Gilliam      ... ... ... ... G.. ... ..T ... ... A.C ... A.. ... ... ... ... A.. T.. ... ...  [ 840]
#EF213091.1_isolate_UT144_strain_Gillium      ... ... ... ... G.. ... ..T ... ... A.C ... A.. ... ... ... ... A.. T.. ... ...  [ 840]
#M63382.1_strain_Kato                         ... ... ... ... ... ... ... ... C.. G.C ... A.. ... ... ... ... A.. ... ... ...  [ 840]
#EF213078.1_isolate_FPW1038_strain_TA716      ... ... ... ... ... ... ... ... ... ... ... ... ... ... ... ... ... ... ... ...  [ 840]
#EF213095.1_isolate_UT302_strainTA763         ... ... ... ... ... ... ... ... ... ... ... ... ... ... ... ... ... ... ... ...  [ 840]
#K13_insert                                   ... ... ... ... ... ... ... ... ... ... ... ... ... ... ... ... ... ... ... ...  [ 840]

#EF213078.1_isolte_UT76_strain_Karp           CCT TAC AAA TAT AAC TGC TCA GGT TGA AGA AGG TAA AGT TAA GGC AGA TTC TGG AGG TAA  [ 900]
#EF213086.1_isolate_UT150_strain_Karp         ... ... ... ... ... ... ... ... ... ... ... ... ... ... ... ... ... ... ... ...  [ 900]
#EF213096.1_isolate_UT125_strain_Gilliam      ... ..G ... ... ..G C.. .G. ... ... ..T ... ... ..G C.. ..T ... ... .A. ... .--  [ 900]
#EF213091.1_isolate_UT144_strain_Gillium      ... ..G ... ... ..G C.. .G. ... ... ..T ... ... ..G C.. ..T ... ... .A. ... .--  [ 900]
#M63382.1_strain_Kato                         ... .G. G.. .G. ..A A.. AG. ... G.. .TC ... ... .-- --- -A. T.G C.. ..A T.C .--  [ 900]
#EF213078.1_isolate_FPW1038_strain_TA716      ... ... ... ... ... ... ... ... ... ... ... ... ... ... ... ... ... ... ... ...  [ 900]
#EF213095.1_isolate_UT302_strainTA763         ... ... ... ... ... ... ... ... ... ... ... ... G.G C.. AAT ..G ... ..A T.C .--  [ 900]
#K13_insert                                   ... ... ... ... ... ... ... ... ... ... ... ... ... ... ... ... ... ... ... ...  [ 900]

#EF213078.1_isolte_UT76_strain_Karp           GAC AAA GGC AGA TTC TGG AGG TGA GAT AAA GGC AGA TTC TGG AGG TGG GAC AGA TGC TCC  [ 960]
#EF213086.1_isolate_UT150_strain_Karp         ... ... ... ... ... ... ... ... ... ... ... ... ... ... ... ... ... ... ... ...  [ 960]
#EF213096.1_isolate_UT125_strain_Gilliam      --- --- --- --- --- --- --- -.. .G. ... ... ... ... ... .T. ... ..T ... ... ...  [ 960]
#EF213091.1_isolate_UT144_strain_Gillium      --- --- --- --- --- --- --- -.. .G. ... ... ... ... ... .T. ... ..T ... ... ...  [ 960]
#M63382.1_strain_Kato                         --- --- --- --- --- --- --- --- --- --- --- -.. .AT .A. .TC ... TG. ... .T. ...  [ 960]
#EF213078.1_isolate_FPW1038_strain_TA716      ... ... ... ... ... ... ... ... ... ... ... ... ... ... ... ... ... ... ... ...  [ 960]
#EF213095.1_isolate_UT302_strainTA763         --- --- -.. TA. .GA .AA CAC ..G T.C GG. T.. .A. .C- --- ... CAA A.. .A- --- -.T  [ 960]
#K13_insert                                   ... ... ... ... ... ... ... ... ... ... ... ... ... ... ... ... ... ... ... ...  [ 960]

#EF213078.1_isolte_UT76_strain_Karp           TAT ACG TAA GCG GTT TAA ACT TAC ACC TCC TCA GCC TAC TAT AAT GCC TAT AAG TAT AGC  [1020]
#EF213086.1_isolate_UT150_strain_Karp         ... ... ... ... ... ... ... ... ... ... ... ... ... ... ... ... ... ... ... ...  [1020]
#EF213096.1_isolate_UT125_strain_Gilliam      ... ... ... ... .CC ... ... ... ... A.. ... ... ... ... ... ... ... ... ... ...  [1020]
#EF213091.1_isolate_UT144_strain_Gillium      ... ... ... ... .CC ... ... ... ... A.. ... ... ... ... ... ... ... ... ... ...  [1020]
#M63382.1_strain_Kato                         ... G.C .C. ... ..A ... ... ... ... A.. ... ... ... ... ... ... ... ... ... T..  [1020]
#EF213078.1_isolate_FPW1038_strain_TA716      ... ... ... ... ... ... ... ... ... ... ... ... ... ... ... ... ... ... ... ...  [1020]
#EF213095.1_isolate_UT302_strainTA763         ..A G.. --- A.C TCC ... ... ... ... G.. ... A.. .GT ... ... ... ... ... ..C ...  [1020]
#K13_insert                                   ... ... ... ... ... ... ... ... ... ... ... ... ... ... ... ... ... ... ... ...  [1020]
```

```
#EF213078.1_isolte_UT76_strain_Karp           TGA TCG TGA CTT TGG GAT TGA TAT TCG TAA CAT ACC TCA GGC GCA AGC --- --- --- ---  [1080]
#EF213086.1_isolate_UT150_strain_Karp         ... ... ... ... ... ... ... ... ... ... ... ... ... ... ... ... --- --- --- ---  [1080]
#EF213096.1_isolate_UT125_strain_Gilliam      ... ... ... TGA G.. .G. ... ..C .GA ..T TC. TG. ... ... TGC T.. T-- --- --- ---  [1080]
#EF213091.1_isolate_UT144_strain_Gillium      ... ... ... TGA G.. .G. ... ..C .GA ..T TC. TG. ... ... TGC T.. T-- --- --- ---  [1080]
#M63382.1_strain_Kato                         G.. ... ... .C. ... .G. ... ... ..C ... .G. ... ... A.G AGG ... TAA TCA CCT GGG  [1080]
#EF213078.1_isolate_FPW1038_strain_TA716      ... ... ... ... ... ... ... ... ... ... ... ... ... ... ... ... --- --- --- ---  [1080]
#EF213095.1_isolate_UT302_strainTA763         ... ... ... TA. G.. TG. ... .G. .AT ... TGA G.. ... A.. T.. ..T A-- --- --- ---  [1080]
#K13_insert                                   ... ... ... ... ... ... ... ... ... ... ... ... ... ... ... ... --- --- --- ---  [1080]

#EF213078.1_isolte_UT76_strain_Karp           TGG GAA TCT TAA TAA --- --- TGA GCA GCG TGC TG- --- --C AGC TAG GAT CGC TTG GTT  [1140]
#EF213086.1_isolate_UT150_strain_Karp         ... ... ... ... ... --- --- ... ... ... ... ..- --- --. ... ... ... ... ... ...  [1140]
#EF213096.1_isolate_UT125_strain_Gilliam      G.. C.. C.A C.G CTT ACT -GT ... ... ... ... ..- --- --. ..A G.. ... T.. ... ...  [1140]
#EF213091.1_isolate_UT144_strain_Gillium      G.. C.. C.A C.G CTT ACT -GT ... ... ... ... ..- --- --. ..A G.. ... T.. ... ...  [1140]
#M63382.1_strain_Kato                         ..A T.. C.. .GG .GC TAA TGA .AT T.G ... ... ..- --- --A C.A ... ... .A. ... ...  [1140]
#EF213078.1_isolate_FPW1038_strain_TA716      ... ... ... ... ... --- --- ... ... ... ... ..- --- --. ... ... ... ... ... ...  [1140]
#EF213095.1_isolate_UT302_strainTA763         CCA C.. G.. C.G C.. AA- --- ... T.C T.T ..T .CG TGG ATT .CG ... ... T.. ... ...  [1140]
#K13_insert                                   ... ... ... ... ... --- --- ... ... ... ... ..- --- --. ... ... ... ... ... ...  [1140]

#EF213078.1_isolte_UT76_strain_Karp           AAA GAA TTG TGC TGG TAT TGA CTA TAG GGT AAA AGA TCC TAA TAA TCC TAA TGG GCC TAT  [1200]
#EF213086.1_isolate_UT150_strain_Karp         ... ... ... ... ... ... ... ... ... ... ... ... ... ... ... ... ... ... ... ...  [1200]
#EF213096.1_isolate_UT125_strain_Gilliam      G.. ... ..A ... ... ... ... ... ..T ... CCC ... ... .C. G.. ... .C. ..- --. ..G  [1200]
#EF213091.1_isolate_UT144_strain_Gillium      G.. ... ..A ... ... ... ... ... ..T ... CCC ... ... .C. G.. ... .C. ..- --. ..G  [1200]
#M63382.1_strain_Kato                         G.. ... ..A ... ... .G. ... ... ..T ... TCC ... ... ... ... ... .C. G.- --. ..G  [1200]
#EF213078.1_isolate_FPW1038_strain_TA716      ... ... ... ... ... ... ... ... ... ... ... ... ... ... ... ... ... ... ... ...  [1200]
#EF213095.1_isolate_UT302_strainTA763         ... AC. G.A ... ... ... ... ... ..T ... G.. G.. ... ... ... ... .-- -.. ..A G..  [1200]
#K13_insert                                   ... ... ... ... ... ... ... ... ... ... ... ... ... ... ... ... ... ... ... ...  [1200]

#EF213078.1_isolte_UT76_strain_Karp           GGT TAT AAA TCC GAT ATT GTT AAA TAT TCC ACA GGG TAA CCC TAT TCC TGT TGG AAA TCA  [1260]
#EF213086.1_isolate_UT150_strain_Karp         ... ... ... ... ... ... ... ... ... ... ... ... ... ... ... ... ... ... ... ...  [1260]
#EF213096.1_isolate_UT125_strain_Gilliam      A.. ... ... ... TG. G.. ... ... ... .A. T.. A.. GC- --- --C A.. .AA C.T .C. G.C  [1260]
#EF213091.1_isolate_UT144_strain_Gillium      A.. ... ... ... TG. G.. ... ... ... .A. T.. A.. GC- --- --C A.. .AA C.T .C. G.C  [1260]
#M63382.1_strain_Kato                         AA. .G. ... ... AG. GC. A.. ... ... ... T.. A.. .C- --- --C G.. .AA ..C ... ..C  [1260]
#EF213078.1_isolate_FPW1038_strain_TA716      ... ... ... ... ... ... ... ... ... ... ... ... ... ... ... ... ... ... ... ...  [1260]
#EF213095.1_isolate_UT302_strainTA763         .A. GG. ... ... TG. G.. ... ... ... ... T.. A.. .C- --- --C G.. ..C .AA T.. ..C  [1260]
#K13_insert                                   ... ... ... ... ... ... ... ... ... ... ... ... ... ... ... ... ... ... ... ...  [1260]

#EF213078.1_isolte_UT76_strain_Karp           GCG AGC ACA GCA GCC TGC AGG GTT -TG CGA TAC ATG ACC ATG AGC AAT GGA GGT ATT TGG  [1320]
#EF213086.1_isolate_UT150_strain_Karp         ... ... ... ... ... ... ... ... -.. ... ... ... ... ... ... ... ... ... ... ...  [1320]
#EF213096.1_isolate_UT125_strain_Gilliam      TA. .C. --- --- -T. G.. .A. A.C T.A AC. ... T.. ... ... GT. .G. ... .AC ... ...  [1320]
#EF213091.1_isolate_UT144_strain_Gillium      TA. .C. --- --- -T. G.. .A. A.C T.A AC. ... T.. ... ... GT. .G. ... .AC ... ...  [1320]
#M63382.1_strain_Kato                         TA. .CA .G- --- CTA ... .AC C.. G.A GT. ... T.A ... ... .T. .C. ... ..C ..C .T.  [1320]
#EF213078.1_isolate_FPW1038_strain_TA716      ... ... ... ... ... ... ... ... -.. ... ... ... ... ... ... ... ... ... ... ...  [1320]
#EF213095.1_isolate_UT302_strainTA763         AA. ... G.- --- CTA ... .AC ... G.. AT. ... T.A .T. ... .T. .C. ... ..C ... .A.  [1320]
#K13_insert                                   ... ... ... ... ... ... ... ... -.. ... ... ... ... ... ... ... ... ... ... ...  [1320]

#EF213078.1_isolte_UT76_strain_Karp           TAA CTG GTC TTG CTG CAT TAT CAA ATG CTA ATA AAC CTA GCG CTT CTC CTG TCA AAG TAT  [1380]
#EF213086.1_isolate_UT150_strain_Karp         ... ... ... ... ... ... ... ... ... ... ... ... ... ... ... ... ... ... ... ...  [1380]
#EF213096.1_isolate_UT125_strain_Gilliam      ..G T.. ..G ..A ... ... .G. ..C ... ... ... ... ... .T. T.A ... ... ... ... ...  [1380]
#EF213091.1_isolate_UT144_strain_Gillium      ..G T.. ..G ..A ... ... .G. ..C ... ... ... ... ... .T. T.A ... ... ... ... ...  [1380]
#M63382.1_strain_Kato                         ..G T.. ..A ..A ... ..A .G. ... ... ... ... ... ... ... T.. ... ..A ... ... ...  [1380]
#EF213078.1_isolate_FPW1038_strain_TA716      ... ... ... ... ... ... ... ... ... ... ... ... ... ... ... ... ... ... ... ...  [1380]
#EF213095.1_isolate_UT302_strainTA763         ..G T.. ..A ... ... ... ... ... ... ... ... ... ... ... ... ... ... ... ... ...  [1380]
#K13_insert                                   ... ... ... ... ... ... ... ... ... ... ... ... ... ... ... ... ... ... ... ...  [1380]

#EF213078.1_isolte_UT76_strain_Karp           TAA GTG ATA AAA TTA CTC AGA TAT ATA GTG ATA TTA AAG CTA TT- GCT GAT ATA GCT GGT  [1440]
#EF213086.1_isolate_UT150_strain_Karp         ... ... ... ... ... ... ... ... ... ... ... .-. ... ... ..T ... ... ... ... ...  [1440]
#EF213096.1_isolate_UT125_strain_Gilliam      ... ... .C. ... ... ..A ... ... ... ... ... .-. .GA .A. ..C ... A.G ... ... AA.  [1440]
#EF213091.1_isolate_UT144_strain_Gillium      ... ... .C. ... ... ..A ... ... ... ... ... .-. .GA .A. ..C ... A.G ... ... AA.  [1440]
#M63382.1_strain_Kato                         ... ... .A. ... ..G TC. ... ... ..C ... ..G .-G ... .CG ..T ... AGA G.. ... ...  [1440]
#EF213078.1_isolate_FPW1038_strain_TA716      ... ... ... ... ... ... ... ... ... ... ... ... ... ... ..- ... ... ... ... ...  [1440]
#EF213095.1_isolate_UT302_strainTA763         ... ... ... ... ... ... ... ... ... ... ... .-. ... .C. ..T ... ... ... ... ...  [1440]
#K13_insert                                   ... ... ... ... ... ... ... ... ... ... ... .-. ... ... ..T ... ... ... ... ...  [1440]

#EF213078.1_isolte_UT76_strain_Karp           ATT GAT GTT CCT GAT GCT GGT TTG CCT AAT AGT GCA ACT GTC GAA CAG ATA CAG AAT AAA  [1500]
#EF213086.1_isolate_UT150_strain_Karp         ... ... ... ... ... ... ... ... ... ... ... ... ... ... ... ... ... ... ... ...  [1500]
#EF213096.1_isolate_UT125_strain_Gilliam      ..C ..A ... ... ... ... CC. ... ... ... ... ... T.. ..T ... ... ... ... GC. ...  [1500]
#EF213091.1_isolate_UT144_strain_Gillium      ..C ..A ... ... ... ... CC. ... ... ... ... ... T.. ..T ... ... ... ... GC. ...  [1500]
#M63382.1_strain_Kato                         ... ..A ... ... AG. .A. CC. ... ... ... ... ... T.. ..T ..G ... ... ... ... ...  [1500]
#EF213078.1_isolate_FPW1038_strain_TA716      ... ... ... ... ... ... ... ... ... ... ... ... ... ... ... ... ... ... ... ...  [1500]
#EF213095.1_isolate_UT302_strainTA763         ... A.. ... ... ... A.. ... ... ... ... ... ..G T.. ... ... ... ... ... .GA ...  [1500]
#K13_insert                                   ... ... ... ... ... ... ... ... ... ... ... ... ... ... ... ... ... ... ... ...  [1500]
```

```
#EF213078.1_isolte_UT76_strain_Karp          ATT GAT GTT CCT GAT GCT GGT TTG CCT AAT AGT GCA ACT GTC GAA CAG ATA CAG AAT AAA  [1500]
#EF213086.1_isolate_UT150_strain_Karp        ... ... ... ... ... ... ... ... ... ... ... ... ... ... ... ... ... ... ... ...  [1500]
#EF213096.1_isolate_UT125_strain_Gilliam     ..C ..A ... ... ... ... CC. ... ... ... ... ... T.. ..T ... ... ... ... GC. ...  [1500]
#EF213091.1_isolate_UT144_strain_Gillium     ..C ..A ... ... ... ... CC. ... ... ... ... ... T.. ..T ... ... ... ... GC. ...  [1500]
#M63382.1_strain_Kato                        ... ..A ... ... AG. .A. CC. ... ... ... ... ... T.. ..T ..G ... ... ... ... ...  [1500]
#EF213078.1_isolate_FPW1038_strain_TA716     ... ... ... ... ... ... ... ... ... ... ... ... ... ... ... ... ... ... ... ...  [1500]
#EF213095.1_isolate_UT302_strainTA763        ... A.. ... ... ... A.. ... ... ... ... ... ..G T.. ... ... ... ... ... .GA ...  [1500]
#K13_insert                                  ... ... ... ... ... ... ... ... ... ... ... ... ... ... ... ... ... ... ... ...  [1500]

#EF213078.1_isolte_UT76_strain_Karp          ATA CAA GAA TTA AAC GAT GTA TTG GAA GAG CTC AGA GAA TCT TTT GAT GGG TAT CTT GGT  [1560]
#EF213086.1_isolate_UT150_strain_Karp        ... ... ... ... ... ... ... ... ... ... ... ... ... ... ... ... ... ... ... ...  [1560]
#EF213096.1_isolate_UT125_strain_Gilliam     ..G G.. ... ... ..T A.. A.C ... ... ... ... ... ... ... ... ... ... ... ... .C-  [1560]
#EF213091.1_isolate_UT144_strain_Gillium     ..G G.. ... ... ..T A.. A.C ... ... ... ... ... ... ... ... ... ... ... ... .C-  [1560]
#M63382.1_strain_Kato                        ..G ... ... ... ..T ... A.. ... ..T ... A.. ... ..T ... ... ..C ... .G. A.. ...  [1560]
#EF213078.1_isolate_FPW1038_strain_TA716     ... ... ... ... ... ... ... ... ... ... ... ... ... ... ... ... ... ... ... ...  [1560]
#EF213095.1_isolate_UT302_strainTA763        ..G ... ... ... ..T ... ... ... ... ..A ... ... ..T G.. ... ... ... ... A.. AA-  [1560]
#K13_insert                                  ... ... ... ... ... ... ... ... ... ... ... ... ... ... ... ... ... ... ... ...  [1560]

#EF213078.1_isolte_UT76_strain_Karp          GGT AAT GCT TTT GCT AAT CAG ATA CAG TTG AAT TTT GTC ATG CCG CAG CAA GCA CAG CAG  [1620]
#EF213086.1_isolate_UT150_strain_Karp        ... ... ... ... ... ... ... ... ... ... ... ... ... ... ... ... ... ... ... ...  [1620]
#EF213096.1_isolate_UT125_strain_Gilliam     --. ... ... ... .T. ... ... ..T ... ..A ... ... CGG ..T ... --- ... ..G ... ..A  [1620]
#EF213091.1_isolate_UT144_strain_Gillium     --. ... ... ... .T. ... ... ..T ... ..A ... ... CGG ..T ... --- ... ..G ... ..A  [1620]
#M63382.1_strain_Kato                        ... ... ... ..C ... ... ... ... ... ... ... ... CG. ..T ... --- ... ... ... ...  [1620]
#EF213078.1_isolate_FPW1038_strain_TA716     ... ... ... ... ... ... ... ... ... ... ... ... ... ... ... ... ... ... ... ...  [1620]
#EF213095.1_isolate_UT302_strainTA763        --. ..C ... ..C .T. G.. ... ... ... ... ... ... ... ... ... .C. ... ... ... ...  [1620]
#K13_insert                                  ... ... ... ... ... ... ... ... ... ... ... ... ... ... ... ... ... ... ... ...  [1620]

#EF213078.1_isolte_UT76_strain_Karp          CAG --- GGG CAA GGG CAG CAA CAG CAA GCT CAA GCT ACA GCG CAA GAA GCA GTA GCA GCA  [1680]
#EF213086.1_isolate_UT150_strain_Karp        ... --- ... ... ... ... ... ... ... ... ... ... ... ... ... ... ... ... ... ...  [1680]
#EF213096.1_isolate_UT125_strain_Gilliam     ... CAG CAA GG. CA. ..A ..G ... ... .GA ... .T. ... ..T ... ..T ... .C. ... ..T  [1680]
#EF213091.1_isolate_UT144_strain_Gillium     ... CAG CAA GG. CA. ..A ..G ... ... .GA ... .T. ... ..T ... ..T ... .C. ... ..T  [1680]
#M63382.1_strain_Kato                        ... GGG CAA GGG CA. ..A ..G ... ... ... ... ... ... ... ... ... ... .C. ..G ...  [1680]
#EF213078.1_isolate_FPW1038_strain_TA716     ... --- ... ... ... ... ... ... ... ... ... ... ... ... ... ... ... ... ... ...  [1680]
#EF213095.1_isolate_UT302_strainTA763        ... CAG ... ... ... ... ... ... ... ... ... ... ... .T. ... ... ... ... ... ...  [1680]
#K13_insert                                  ... --- ... ... ... ... ... ... ... ... ... ... ... ... ... ... ... ... ... ...  [1680]

#EF213078.1_isolte_UT76_strain_Karp          GCA GCT GTT AGG CTT TTA AAT GGC AAT AAT CAG ATT GAG CAG TTA TAT AGA GAT CTT GTT  [1740]
#EF213086.1_isolate_UT150_strain_Karp        ... ... ... ... ... ... ... ... ... ... ... ... ... ... ... ... ... ... ... ...  [1740]
#EF213096.1_isolate_UT125_strain_Gilliam     ... ... ... ... GC. ... ... ..T ... G.A ... ... ATA ... ... ... .A. ... ... ...  [1740]
#EF213091.1_isolate_UT144_strain_Gillium     ... ... ... ... GC. ... ... ..T ... G.A ... ... ATA ... ... ... .A. ... ... ...  [1740]
#M63382.1_strain_Kato                        ... ... ... ... G.. ... ... AA. ... G.. ... ... ATA A.. ... ... .A. ... ... ...  [1740]
#EF213078.1_isolate_FPW1038_strain_TA716     ... ... ... ... ... ... ... ... ... ... ... ... ... ... ... ... ... ... ... ...  [1740]
#EF213095.1_isolate_UT302_strainTA763        ... ... ... ... ... ... ... ... ... G.. ... ... .T. ... ... ... .A. ... ... ...  [1740]
#K13_insert                                  ... ... ... ... ... ... ... ... ... ... ... ... ... ... ... ... ... ... ... ...  [1740]

#EF213078.1_isolte_UT76_strain_Karp          AAA TTG CAG CGT CAT GCA GGA ATT AAG AAA GCG ATG GAA AAA TTA GCT GCC CAA CAA GAA  [1800]
#EF213086.1_isolate_UT150_strain_Karp        ... ... ... ... ... ... ... ... ... ... ... ... ... ... ... ... ... ... ... ...  [1800]
#EF213096.1_isolate_UT125_strain_Gilliam     ... ... ... ... ... ... ... ... .G. ..G ..T ... ... C.. ..G ... ... ... G.. ...  [1800]
#EF213091.1_isolate_UT144_strain_Gillium     ... ... ... ... ... ... ... ... .G. ..G ..T ... ... C.. ..G ... ... ... G.. ...  [1800]
#M63382.1_strain_Kato                        ... ... A.. ... ... ... ... ... ..A ... ..T ... ... G.. ..G ... ..T ... G.C .G.  [1800]
#EF213078.1_isolate_FPW1038_strain_TA716     ... ... ... ... ... ... ... ... ... ... ... ... ... ... ... ... ... ... ... ...  [1800]
#EF213095.1_isolate_UT302_strainTA763        ... ... ... ... ... ... ... ... ... ... ... ... ... ... ... ... ... ... ... ...  [1800]
#K13_insert                                  G-- --- --- --- --- --- --- --- --- --- --- --- --- --- --- --- --- --- --- ---  [1800]

#EF213078.1_isolte_UT76_strain_Karp          GAA GAT GCA AAG AAT CAA GGT GAA GGT GAC TGC AAG CAG CAA CAA GGA GCA TCT GAA AAA  [1860]
#EF213086.1_isolate_UT150_strain_Karp        ... ... ... ... ... ... ... ... ... ... ... ... ... ... ... ... ... ... ... ...  [1860]
#EF213096.1_isolate_UT125_strain_Gilliam     .GT ... .AT C.A ... ... ..A AGT T.C A.G GAT ... A.. ..G ... AT. ... A.. ... G.C  [1860]
#EF213091.1_isolate_UT144_strain_Gillium     .GT ... .AT C.A ... ... ..A AGT T.C A.G GAT ... A.. ..G ... AT. ... A.. ... G.C  [1860]
#M63382.1_strain_Kato                        .GT TG. AAT --- --- --- ..A .GT ... ..T AAT ... A.. A.G .G. ... ... ... ... G.C  [1860]
#EF213078.1_isolate_FPW1038_strain_TA716     ... ... ... ... ... ... ... ... ... ... ... ... ... ... ... ... ... ... ... ...  [1860]
#EF213095.1_isolate_UT302_strainTA763        ... ... ..C ... ... ... ... ... ... ... ... ... ... ... ... ... ... ... ... ...  [1860]
#K13_insert                                  --- --- --- --- --- --- --- --- --- --- --- --- --- --- --- --- --- --- --- ---  [1860]
```

Figure S1. Multiple DNA alignment of 56-kDa type-specific antigen (TSA) gene among varied strains of *O. tsutsugamushi*, i.e., Karp UT76 strain (EF213078.1), Karp UT150 strain (EF213086.1), Gilliam UT125 strain (EF213096.1), Gilliam UT144 strain (EF213091.1), Kato strain (M63382.1), TA716 strain (EF213078.1), TA763 strain (EF213095.1), and K13 clone containing partial gene derived from Karp serotype.
